# Influences of (in)congruences in psychological entitlement and felt obligation on ethical behavior

**DOI:** 10.3389/fpsyg.2022.1052759

**Published:** 2023-01-09

**Authors:** Qin Chen, Yifei Shen, Li Zhang, Zhenduo Zhang, Junwei Zheng, Jing Xiu

**Affiliations:** ^1^School of Management, Harbin Institute of Technology, Harbin, China; ^2^School of Economics and Management, Dalian University of Technology, Dalian, China; ^3^Faculty of Civil Engineering and Mechanics, Kunming University of Science and Technology, Kunming, China; ^4^College of Applied Economics, University of Chinese Academics of Social Sciences, Beijing, China

**Keywords:** felt obligation, psychological entitlement, work engagement, helping behavior, unethical behavior

## Abstract

**Introduction:**

Psychological entitlement and felt obligation are two correlated but distinctive conceptions. Prior studies have mainly explored their influences on employees' (un)ethical behavior, respectively. Recently, several studies suggest the interactive impacts of psychological entitlement with felt obligation on individual behavioral choices. In consistency with these studies, the present study focuses on the influences of (in)congruences in psychological entitlement and felt obligation on employees' (un)ethical behavior.

**Methods:**

A two-wave multi-source questionnaire survey is conducted to collect 202 matched questionnaires from full-time Chinese workers. The polynomial regression with response surface analysis is employed to test hypotheses.

**Results:**

The results indicate that: (1) employees have higher levels of work engagement and helping behavior but lower levels of unethical behavior when their psychological entitlement and felt obligation are balanced at higher levels rather than lower levels; (2) employees have higher levels of work engagement and helping behavior but lower levels of unethical behavior when they have higher levels of felt obligation but lower levels of psychological entitlement compared to those having lower levels of felt obligation but higher levels of psychological entitlement; and (3) work engagement mediates the relationship between (in)congruences in psychological entitlement and felt obligation and employees' helping behavior and unethical behavior.

**Discussion:**

This study provides a novel insight into the interactive influences of (in)congruence in psychological entitlement and felt obligation on employees' ethical behavioral choices.

## 1. Introduction

Psychological entitlement and felt obligation are two basic psychological characteristics of Generation Y employees (Anderson et al., 2017). Prior studies have regarded psychological entitlement and felt obligation as two contradictory conceptions, highlighting different influences of psychological entitlement and felt obligation on employees' ethical behavior. For instance, psychological entitlement facilitates deviant behavior and workplace bullying but impedes organizational citizenship behavior (OCB; Qin et al., 2020). In contrast, felt obligation motivates employees to engage in OCB but mitigates their intention to conduct counterproductive behavior and aggressive behavior (Moorman and Harland, 2002; Chen et al., 2021).

**Figure 1:**
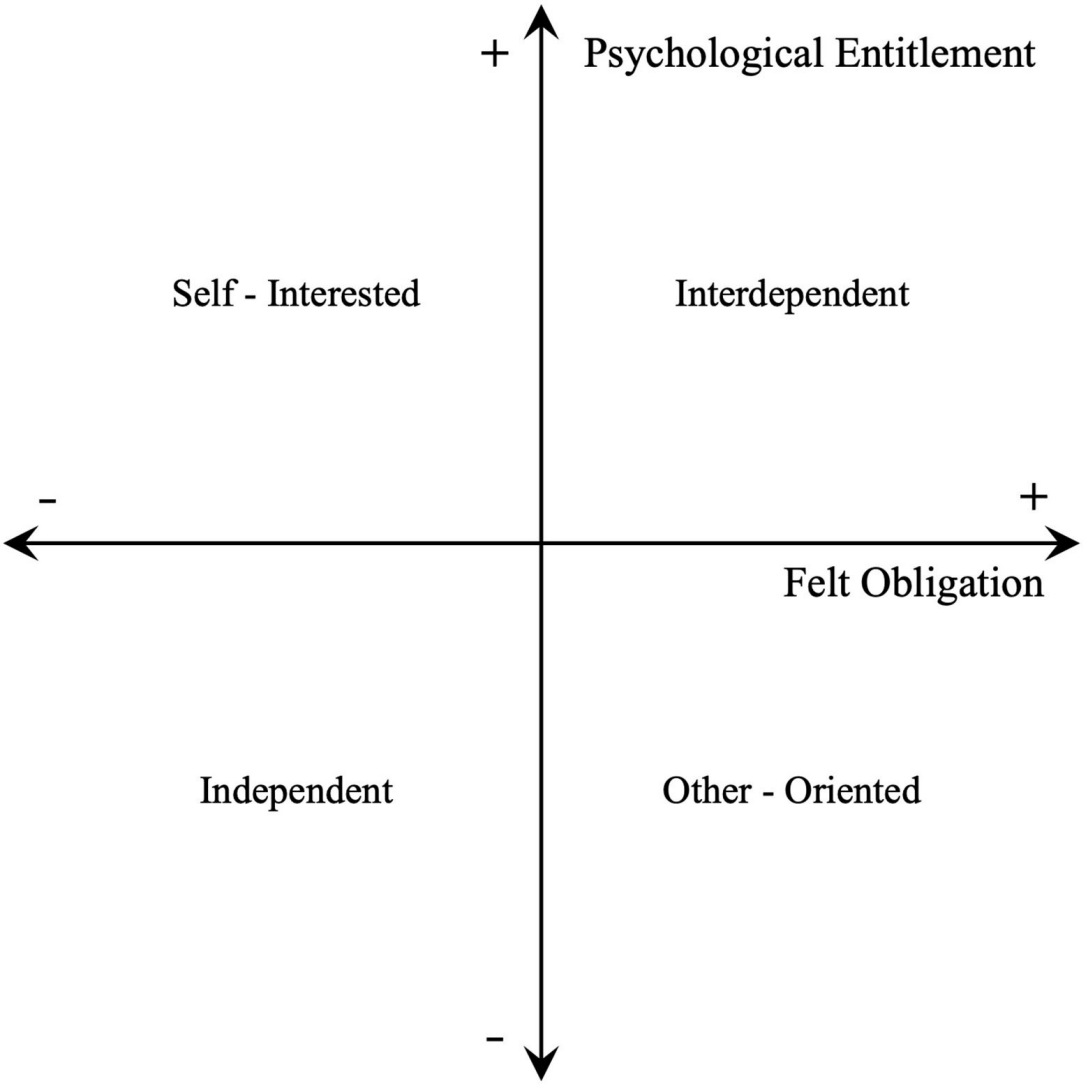
Felt obligation and psychological entitlement social orientations.

Entitlement is the extent to which employees believe that they deserve rewards and appreciation from organizations (Campbell et al., 2004). Therefore, entitlement is a typical self-interested personality. On the other hand, obligation is the degree to which employees believe that they owe consideration and resources to society (Eisenberger et al., 2001). Obligation is developed as tendencies to deviate from self-interests and is defined as an other-oriented personality. Brummel and Parker (2015) believed that psychological entitlement and felt obligation are not the two ends on the same self-interests continuum but are better conceptualized as two distinct theoretical constructs. These two personality traits shape individuals' behavior coordinately. They highlight the importance of studying the self-interested trait (i.e., psychological entitlement) in conjunction with the prosocial trait (i.e., felt obligation) because the absence of self-interest may not represent prosocial tendencies. On the basis of these arguments, they propose the orthogonal structure of felt obligation and psychological entitlement and demonstrate the underlying theoretical lens to explain how these two traits impact employees' behavioral choices (refer to Figure 1).

Running along the line from independent to interdependent continuum, employees' behavioral choices can be explained by equity sensitivities based on the equity theory (Mowday, 1991). Entitled employees are sensitive to the ratio of inputs to outputs at workplaces and they are eager to differentiate themselves from their peer coworkers (Lange et al., 2019), while felt obligation motivates employees to devote time and resources to their jobs and to perceive their rewards fairly (Brummel and Parker, 2015) and mitigates the high sensitivity to the ratio of inputs to outputs driven by psychological entitlement. Moreover, entitled and obligated employees tend to distinguish themselves from their coworkers and categorize themselves as professionals with specialized knowledge about their jobs by contributing more to the organizational effectiveness compared with their peers. Therefore, employees tend to exhibit more work efforts and prosocial behavior when psychological entitlement and felt obligation are balanced at higher levels rather than lower levels (Tennent, 2021).

Running along the line from self-interested to other-oriented continuum, social value orientation based on the social interdependence theory is used to explain behavioral tendencies (Van Lange, 1999). Employees with high psychological entitlement but low felt obligation are self-serving and neglect their coworkers' benefits (Eissa and Lester, 2022). They tend to engage in more deviant behavior which is disadvantageous to organizational effectiveness (Naseer et al., 2020). By contrast, employees with high felt obligation but low psychological entitlement are other-oriented. They are more likely to exhibit OCB and focus on their tasks to contribute to organizational effectiveness (Roch et al., 2019). Therefore, employees tend to be good citizens in organizations with low psychological entitlement but high felt obligation rather than high psychological entitlement but low felt obligation.

Although Brummel and Parker (2015) have proposed the orthogonal structure of felt obligation and psychological entitlement, recent studies are still trying to explore the influences of these two traits on employees' work behavior separately. Less is known about the underlying mechanism through which the (in)congruences in psychological entitlement and felt obligation impact employees' (un)ethical behavior (i.e., helping behavior and unethical behavior). Helping behavior denotes voluntary assistance to others in accomplishing their goals or preventing the occurrence of problems (Yue et al., 2017). In contrast, unethical behavior means violating social norms for moral behavior, such as pilfering company materials, giving gifts/favors in exchange for preferential treatment, and divulging confidential information (Paterson and Huang, 2019). Helping behavior and unethical behavior are the two typical aspects of ethical behavior in organizational psychological research (Miao et al., 2020; Li, 2021). Therefore, this study will adopt these two variables as outcomes of the (in)congruences in psychological entitlement and felt obligation. In addition, this study will employ work engagement, denoted as a positive, fulfilling work-related state of mind that is characterized by vigor, dedication, and absorption, as a mediator. Work engagement has been used by prior studies to explain the inner path linking personalities and (un)ethical behavioral choices (Bakker et al., 2012). In addition, work engagement has also been adopted as an indicator of perceptions of equity and prosocial orientations in organizations (Agarwal, 2014; Gheorghe et al., 2022).

**Figure 2:**
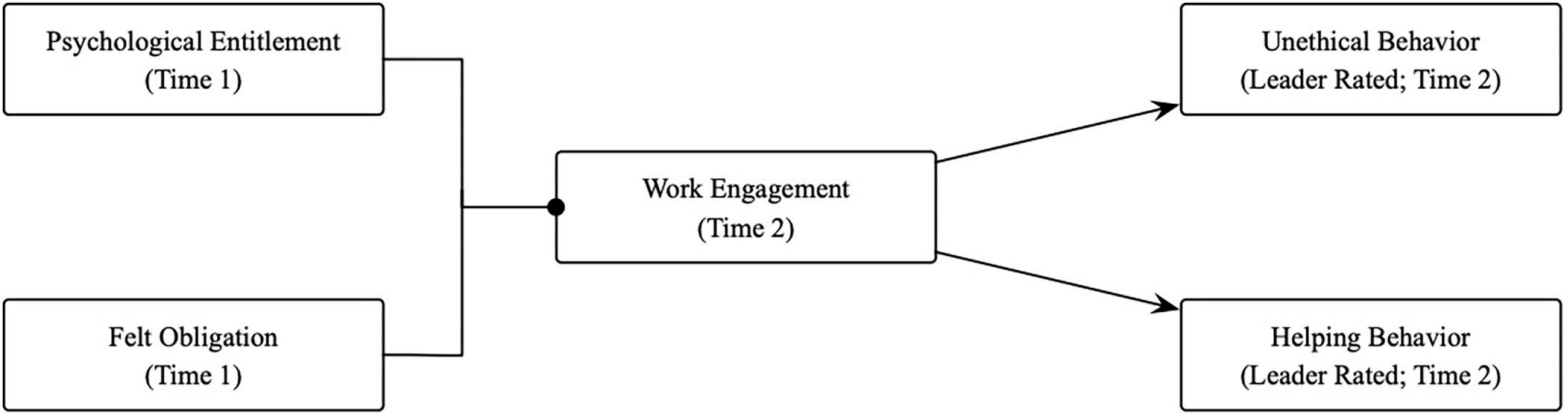
The conceptual model.

Based on the equity theory and the social interdependence theory, the conceptual model is proposed (refer to Figure 2). This study intends to collect data from full-time Chinese workers to test the conceptual model using a multi-source two-wave questionnaire design. By doing so, this study will contribute to literature both on psychological entitlement and on felt obligation. First, we challenge the consensus that felt obligation is beneficial while psychological entitlement is detrimental, emphasizing the need to simultaneously consider psychological entitlement and felt obligation. Previous research has mainly examined the influences of psychological entitlement and felt obligation on (un)ethical behavior separately, reaching a consensus that felt obligation promotes pro-social behavior while psychological entitlement fosters unethical behavior (Lee et al., 2019b; Miao et al., 2020). This line of research fails to shed light on the interactive influences of felt obligation and psychological entitlement on individuals' (un)ethical behavioral decision-making, which has been addressed by Tennent (2021). We provided a novel perspective to investigate how (in)congruences in psychological entitlement and felt obligation influence employees' (un)ethical behavior by adopting polynomial regression with response surface analysis. Second, this study identifies work engagement as the underlying mechanism linking psychological entitlement-felt obligation fit with (un)ethical behavior. The importance of work engagement is emphasized in both the equity theory and the social interdependence theory. In the equity theory, work engagement is the proximal outcome of the sense of equity and further motivates individuals to perform ethical behavior (Cao et al., 2020). In the social interdependence theory, other-oriented rather than self-oriented employees are more likely to devote resources to engage in their jobs and to contribute to the organizational effectiveness by inhibiting unethical behavior but fostering ethical behavior (Gheorghe et al., 2022). This study adopts work engagement as the mediator in the relationship between (in)congruences in psychological entitlement and felt obligation, and (un)ethical behavior contributing to both the equity theory and the social interdependence theory.

## Hypothesis development

### Psychological entitlement and felt obligation

It is a pervasive sense that an individual feels they deserve more than others, even if this is not commensurate with one's actual abilities and efforts (Zitek and Jordan, 2021). The concept of psychological entitlement is derived from narcissistic personality (Lee et al., 2019a). However, recent research has differentiated psychological entitlement from narcissism. Psychologists argue that narcissism is primarily about the self. By contrast, psychological entitlement is mainly about the self in relation to others (Lee et al., 2019a). To keep a superior status compared to peers, entitled employees are more likely to engage in unfavorable actions at work. Prior studies have explored the positive relationship between psychological entitlement and unethical pro-organizational behavior, aggressive behavior, workplace incivility, and workplace deviance based on the social identity theory, equity theory, and social exchange theory (Lee et al., 2019b; Liu and Zhou, 2021). Moreover, the literature indicates that ethical leadership and organizational justice play key roles in inhibiting the positive relationship between psychological entitlement and ethical behavior (Al Halbusi et al., 2021a, 2022b; Al Halbusi, 2022).

Felt obligation and psychological entitlement are the two basic psychological characteristics of Generation Y employees (Anderson et al., 2017). The concept of felt obligation is developed by Eisenberger et al. (2001) based on the research of perceived organizational support. Felt obligation is a prescriptive belief regarding whether one should care about the organization's wellbeing and should help the organization reach its goals (Ogunfowora et al., 2021). Contrary to psychological entitlement, felt obligation is a typical prosocial trait and has always been regarded as an antecedent to ethical behavior at work. Prior studies have explored the positive influences of felt obligation on helping behavior, green behavior, and voice behavior based on the social exchange theory and the social identity theory (Eisenberger et al., 2001; Campbell et al., 2004; Al Halbusi et al., 2022a). Research also suggests that ethical leadership and organizational justice are important in fostering felt obligation and promoting ethical behavior (Al Halbusi et al., 2021b; Halbusi et al., 2021).

### Differentiating psychological entitlement congruences from felt obligation congruences

Equity sensitivity is one of the core concepts in the equity theory, which is referred to as the degree to which people respond to situations of perceived inequality due to their preferences for equality (Miles et al., 1994). Entitled employees prefer to undermine their rewards compared with their devotion relative to their colleagues (Li, 2021). By contrast, obligation motivates employees to behave altruistically. They tend to give more than they have received compared with their coworkers (Kim and Qu, 2020). Obligation would mitigate the high sensitivity to equity stimulated by entitlement. Moreover, Tennent (2021) suggested that in social interactions, obligated and entitled employees are more likely to engage in helping behavior to categorize themselves as professional members.

Alongside the congruence line, employees with high entitlement and high obligation are labeled as interdependent. They care about both their rewards and organizational effectiveness. The equity theory indicates that employees are motivated to seek perceptions of equity in organizations (Kollmann et al., 2020). Entitled employees are chasing more rewards and higher status in comparison with their coworkers. They prefer to compare the inputs and outputs at work with those of their coworkers (Brummel and Parker, 2015). On the other hand, high-level obligation drives them to take organizations' benefits into consideration simultaneously when they are acquiring their benefits (Brant and Castro, 2019; Lorinkova and Perry, 2019). They are more likely to engage in their work to achieve higher performance and behave pro-socially and legitimately to make themselves distinguishable and enhance their sense of equity (Tennent, 2021). By contrast, employees with low entitlement and low obligation are labeled as independent. They are indifferent to their benefits or the organizational development. Due to the low obligation and low entitlement, they are less likely to engage in prosocial behavior to benefit the organizations and coworkers (Thompson et al., 2020). Worse still, they will not devote their resources to fully fulfill their work roles and maintain high-level performance (Xu et al., 2020). Accordingly, this study proposed the following hypothesis:

*H1: When an employee's psychological entitlement is aligned with felt obligation at a higher level rather than a lower level, employees tend to exhibit a higher level of work engagement (H1a) and helping behavior (H1b), but a lower level of unethical behavior (H1c)*.

### 2.3. Differentiating psychological entitlement incongruences from felt obligation incongruences

Social value orientation, developed from the social interdependence theory, is denoted as the weights people assign to their own and others' outcomes in situations of interdependence (Balliet et al., 2009). Employees with high felt obligation are motivated to cooperate with others, engage in their current jobs, and help coworkers rather than damage their benefits. Psychological entitlement is a subdimension of having a narcissistic personality (Ackerman et al., 2019). Prior studies suggest that entitled individuals have a sustained inflated view of themselves (Lange et al., 2019). The proximal behavioral results of such negative emotional states include aggressive behavior in the workplace, such as interpersonal deviance, incivility, and bullying (Vatankhah and Raoofi, 2018; Naseer et al., 2020).

Alongside the asymmetry line, employees with high psychological entitlement and low felt obligation are categorized as self-oriented. They are more likely to serve themselves by damaging others' benefits (Vatankhah and Raoofi, 2018). The social interdependence theory divides employees into three categories: prosocial, individualistic, and competitive (Johnson and Johnson, 2005). Entitled employees with low felt obligation are more likely categorized as individualistic and competitive (Vatankhah and Raoofi, 2018). Existing studies demonstrate that they are less engaged in their work (Thompson et al., 2020), tend to behave unethically (Miao et al., 2020), and are disinclined to behave helpfully (Li et al., 2022). By contrast, employees with high felt obligation are categorized as other-oriented or prosocial. Their behavior is motivated by felt obligation rather than entitlement. According to the predominant obligation literature, they prefer to devote their resources to current jobs (Ackerman et al., 2019), exhibit extra-role behavior to facilitate organizational effectiveness (Roch et al., 2019), and not engage in unethical behavior (Gheorghe et al., 2022). Therefore, this study proposed the second hypothesis:

*H2: Employees with higher felt obligation but lower psychological entitlement have a higher level of work engagement (H2a) and helping behavior (H2b) but a lower level of unethical behavior (H2c) compared with employees with higher psychological entitlement but lower felt obligation*.

### 2.4. Work engagement as a mediator of the (in)congruence effect on helping behavior and unethical behavior

Furthermore, it is assumed that employees' work engagement mediates the psychological entitlement-felt obligation (in)congruence effect of their helping behavior and unethical behavior. Prior studies supply fruitful evidence for the influences of work engagement on both helping behavior and unethical behavior based on both the equity theory and the social independence theory (Sulea et al., 2012; Meynhardt et al., 2020). Employees who are psychologically engaged in their work have a greater likelihood of performing things beyond job requirements and devoting more time and effort to work-related issues and relationships, which equals helping behavior (Mostafa, 2019). Moreover, work engagement provides employees with self-control resources to regulate their behavior, which contributes to the mitigation of unethical behavior. As noted above, work engagement is shaped by psychological entitlement and felt obligation jointly, which in turn will influence employees' helping behavior and unethical behavior. Therefore, we hypothesized as follows:

*H3: An employee's work engagement mediates the relationship between (in)congruence in psychological entitlement and felt obligation, and the employee's helping behavior (H3a) and unethical behavior (H3b)*.

## 3. Methods

### 3.1. Procedures and participants

To avoid common method bias (CMB; Podsakoff et al., 2003) and social desirability bias (SDB; Nederhof, 1985), this study adopted a two-wave multi-source questionnaire survey design. We collected data from two subsidiary companies of a construction group company located in Beijing, China. We contacted the human resource managers of the two subsidiary companies and acquired their assistance. Before the questionnaire survey, we have an interview with human resource managers and frontline employees to confirm the clarity, readability, comprehension, and suitability of our questionnaires (Al Halbusi et al., 2021a). Then, with the aid of the two human resource managers, we sent emails through their intra-company information systems. In the email, we elaborated on the research purpose and survey process. We recruited participants for our samples and asked for their consent to participate. The respondents were assured that their responses were confidential and that they had the right to end participation in the survey at any time. We formed two research groups in WeChat, a universal social media application in China, and invited the respondents to join the WeChat groups. The research assistants distributed questionnaires through mobile websites, with the questionnaires completed through mobile phones in WeChat groups. Before the data collection, online informal consent was secured from the respondents.

In the first wave, the respondents were required to assess their demographic information, psychological entitlement, and felt obligation. Totally, 227 participants completed this survey. In the second wave, the respondents were required to assess their work engagement. We contacted their team leaders and required them to assess the specific participants' helping behavior and unethical behavior. Finally, 202 participants with their leaders completed the survey in this wave. The effective response rate was 88.98%. Among the samples, 48.5 were women; 62.9% held bachelor's degrees and 17.8% held master's degrees or above; 61.4% were married; the average age of the employees was 32.32 (±6.33); and the average tenure in their companies was 5.83 years (±5.30). A drop-out analysis was conducted, which found that the dropped samples had no differences in demographic information from the completed samples. The participants were notified that when they completed the first wave of the questionnaire, they would receive RMB 15 (≈USD 2.09); and when they completed both parts of the questionnaire, they would receive RMB 50 (≈USD 6.95). The high reward for completing both parts of the questionnaire was adopted to ensure an effective response rate.

### 3.2. Measures

The original questionnaires were published in English. A back-to-back translation procedure was adopted to ensure translation accuracy (Brislin, 1980). A 5-point Likert scale was employed, with “1” indicating “strongly disagree” and “5” indicating “strongly agree.” The measured variables are as follows:

#### 3.2.1. Psychological entitlement

Psychological entitlement was assessed using a 4-item scale, adapted from Yam et al. (2014). A sample item was written as “I honestly feel I'm just deserving more than others.” Cronbach's alpha of this scale was 0.91.

#### 3.2.2. Felt obligation

It was assessed using a 6-item scale developed by Eisenberger et al. (2001). A sample item was written as “I feel a personal obligation to do whatever I can to help the organization achieve its goals.” Cronbach's Alpha of this scale was 0.87.

#### 3.2.3. Work engagement

The three-item ultra-short work engagement developed by Schaufeli and De Witte (2017) was adopted in this study. The sample item was “At my work, I feel bursting with energy.” This scale yielded Cronbach's Alpha of 0.82.

#### 3.2.4. Unethical behavior

The five-item scale developed by Paterson and Huang (2019) was preferred in this study. The sample item was “The employee does personal business during company time.” Cronbach's Alpha of this scale was 0.81.

#### 3.2.5. Helping behavior

The three-item scale proposed by Yue et al. (2017) was utilized in this study. The sample item was “The employee helps other employees when it is clear that their workload is too heavy.” This scale yielded Cronbach's Alpha of 0.77.

#### 3.2.6. Control variables

Considering the influences of demographic information on (un)ethical behavior, this study controlled gender, age, and education in the regression analysis, in accordance with previous studies (Savir and Gamliel, 2019).

## 4. Results

### 4.1. Analytical strategy

Polynomial regression with response surface analysis was adopted to test the abovementioned hypotheses. This method has been used in psychological and management studies to explore how the combination of two independent variables impacts other dependent variables, particularly in the case of congruence and discrepancy measures (Edwards, 1994). Polynomial regression with response surface analysis can provide a three-dimensional view of the joint influences of two independent predictors on one outcome which makes this statistical approach superior to other traditional regression analyses (Edwards and Parry, 1993).

The classical equation for polynomial regression was *Z* = b_0_ + b_1_*X* + b_2_*Y* + b_3_*X*^2^ + b_4_*XY* + b_5_*Y*^2^ + e. In this equation, *Z* referred to the dependent variables (work engagement, helping behavior, and unethical behavior), *X* represented psychological entitlement, and *Y* represented felt obligation. In the response surface analysis, coefficients in the polynomial regression were used to examine the surface pattern, which could provide a three-dimensional visual representation of the data for the interpretation of the polynomial regression results. The surface pattern was determined by the slope and curvature of the congruence line (*X* = *Y*) and the incongruence line (*X* = –*Y*) (Edwards and Cable, 2009).

Before polynomial regression with response surface analysis, *X* and *Y* were cantered (Edwards, 1994). To test hypothesis 1, it was determined whether the slope along the congruence line (*X* = *Y*) was significantly positive for the outcomes consisting of work engagement and helping behavior, and significantly negative for the outcome consisting of unethical behavior. To test hypothesis 2, it was determined whether the slope along the incongruence line (*X* = –*Y*) was significantly negative for the outcomes such as work engagement and helping behavior, and significantly positive for the outcome such as unethical behavior. To test hypothesis 3, the block approach proposed by Edwards and Cable (2009) was adopted. A block variable that combined the five polynomial terms was calculated based on their respective weights in the polynomial regression analysis. Afterward, path analysis was conducted to examine the mediation model using Mplus 7.4.

### 4.2. Confirmatory factor analysis

Before implementing the regression analysis, we conducted confirmatory factor analysis (CFA) to test the survey validity. The results in Table 1 indicated that the proposed 5-factor model [ $\chi_{\left(199\right)}^{2}$ = 357.41, CFI = 0.94, TLI = 0.93, RMSEA = 0.06, RMR = 0.085] had a better fit than other models (χ^2^ ≥ 524.33, *p* < 0.01).

**Table 1:** Results of confirmatory factor analysis.

**Models**	**Factors**	**χ^2^**	**DF**	**χ^2^/DF**	**Δχ^2^**	**RMSEA**	**CFI**	**TLI**	**RMR**
5-factor model	PE, FO, WE, UB, HB	357.41	199	1.80		0.06	0.94	0.93	0.05
4-factor model	PE + FO, WE, UB, HB	881.74	203	4.34	524.33^**^	0.13	0.76	0.72	0.13
3-factor model	PE + FO, WE, UB + HB	1,051.36	206	5.10	693.94^**^	0.14	0.70	0.66	0.14
2-factor model	PE + FO + WE, UB + HB	1,239.08	208	5.96	881.67^**^	0.16	0.63	0.59	0.14
1-factor model	PE + FO + WE + UB + HB	1,558.10	209	7.46	1,200.69^**^	0.18	0.51	0.46	0.13

^**^p < 0.01; N = 202. PE, psychological entitlement; FO, felt obligation; WE, work engagement; UB, unethical behavior; HB, helping behavior.

### 4.3. Descriptive statistics and correlation analysis

We also calculated the means and standard deviations for the different variables. Those correlations between focal variables are shown in Table 2.

**Table 2:** Results of descriptive statistics and correlation analysis.

	**1**	**2**	**3**	**4**	**5**	**6**	**7**	**8**
1. Gender								
2. Age	0.04							
3. Education	0.20^**^	0.06						
4. Helping behavior	−0.01	0.08	−0.03	(0.77)				
5. Unethical behavior	0.01	0.01	−0.10	−0.27^**^	(0.81)			
6. Work engagement	−0.14^*^	0.03	−0.07	0.38^**^	−0.31^**^	(0.82)		
7. Psychological entitlement	0.00	−0.10	−0.08	−0.11	0.46^**^	−0.02	(0.91)	
8. Felt obligation	−0.05	0.00	0.07	0.46^**^	−0.57^**^	0.44^**^	−0.30^**^	(0.87)
Mean	1.52	32.34	1.98	3.93	2.27	3.71	2.54	4.06
SD	0.50	6.35	0.61	0.57	0.80	0.65	0.94	0.62

^**^ p < 0.01, ^*^p < 0.05; N = 202; Values in the parentheses are the Cronbach's alpha.

### 4.4. Polynomial regression with response surface analysis

We then conducted a polynomial regression analysis using SPSS v.21.0 to test the hypotheses. The results of Model 2 (Table 3) indicated that psychological entitlement was not significantly associated with work engagement (*B* = 0.08, SE = 0.05, n.s.) but felt obligation was significantly positively associated with work engagement (*B* = 0.50, SE = 0.07). To test H1 and H2, we used response surface analysis and examined the response surface pattern based on the curvature and slopes of the congruence and incongruence lines. The results of Model 2 in Table 3 showed that the slope (β = 0.58, SE = 0.10, *p* < 0.01) for the congruence line (*x* = *y*) was positive and significant. The significantly positive slope indicated that, compared with independent employees, those interdependent employees had a higher level of work engagement, thus verifying H1a. The results of Model 2 in Table 3 showed that the slope (β = −0.43, SE = 0.08, *p* < 0.01) for the incongruence line (*x* = –*y*) was significant. This result indicated that the level of work engagement was higher for other-oriented employees than for self-oriented employees, thereby supporting H2a.

**Table 3:** Results of polynomial regression with response surface analysis.

	**Work engagement**				**Helping behavior**				**Unethical behavior**			
	**Model 1**		**Model 2**		**Model 3**		**Model 4**		**Model 5**		**Model 6**	
	***B***	**SE**	***B***	**SE**	***B***	**SE**	***B***	**SE**	***B***	**SE**	***B***	**SE**
Constant	3.95	0.29	1.88	0.46	3.75	0.26	1.83	0.41	2.41	0.36	3.87	0.49
Gender	−0.18	0.09	−0.16	0.08	0.00	0.08	0.03	0.08	0.05	0.12	0.01	0.09
Age	0.00	0.01	0.00	0.01	0.01	0.01	0.01	0.01	0.00	0.01	0.01	0.01
Education	−0.04	0.08	−0.10	0.07	−0.03	0.07	−0.06	0.06	−0.14	0.10	−0.05	0.08
Psychological entitlement (*X*)			0.08	0.05			0.03	0.04			0.30^**^	0.05
Felt obligation (*Y*)			0.50^**^	0.07			0.45^**^	0.06			−0.60^**^	0.08
*X* ^2^			−0.08	0.04			−0.02	0.03			−0.01	0.04
*X* × *Y*			0.00	0.08			−0.05	0.07			−0.16	0.08
*Y* ^2^			−0.12	0.09			0.05	0.08			−0.08	0.10
*F*	1.53		7.79^**^		0.50		6.93^**^		0.78		11.84^**^	
*R* ^2^	0.02		0.25		0.01		0.23		0.01		0.43	
Δ*R*^2^			0.23^**^				0.22^**^				0.42^**^	
Slope along *x* = *y* (as related to *Z)*			0.58^**^	0.10			0.48^**^	0.09			−0.31^**^	0.10
Curvature on *x* = *y* (as related to *Z*)			−0.20	0.13			−0.02	0.12			−0.25	0.14
Slope along *x* = –*y* (as related to *Z*)			−0.43^**^	0.08			−0.42^**^	0.07			0.90^**^	0.08
Curvature on *x* = –*y* (as related to *Z*)			−0.20	0.14			0.08	0.12			0.07	0.15

^**^p < 0.01; N = 202.

The results of Model 4 (Table 3) demonstrated that psychological entitlement was not significantly associated with work engagement (*B* = 0.03, SE = 0.04, n.s.) but felt obligation was significantly positively associated with work engagement (*B* = 0.45, SE = 0.06). The results of Model 4 in Table 3 showed that the slope (β = 0.48, *SE* = 0.09, *p* < 0.01) for the congruence line (*x* = *y*) was positive and significant. The significantly positive slope indicated that, compared with independent employees, those interdependent employees had a higher level of helping behavior, thereby supporting H2b. The results of Model 4 in Table 3 showed that the slope (β = −0.42, *SE* = 0.07, *p* < 0.01) for the incongruence line (*x* = –*y*) was significant. This result indicated that the level of helping behavior was higher for other-oriented employees than for self-oriented employees, thus confirming H2b.

**Figure 3:**
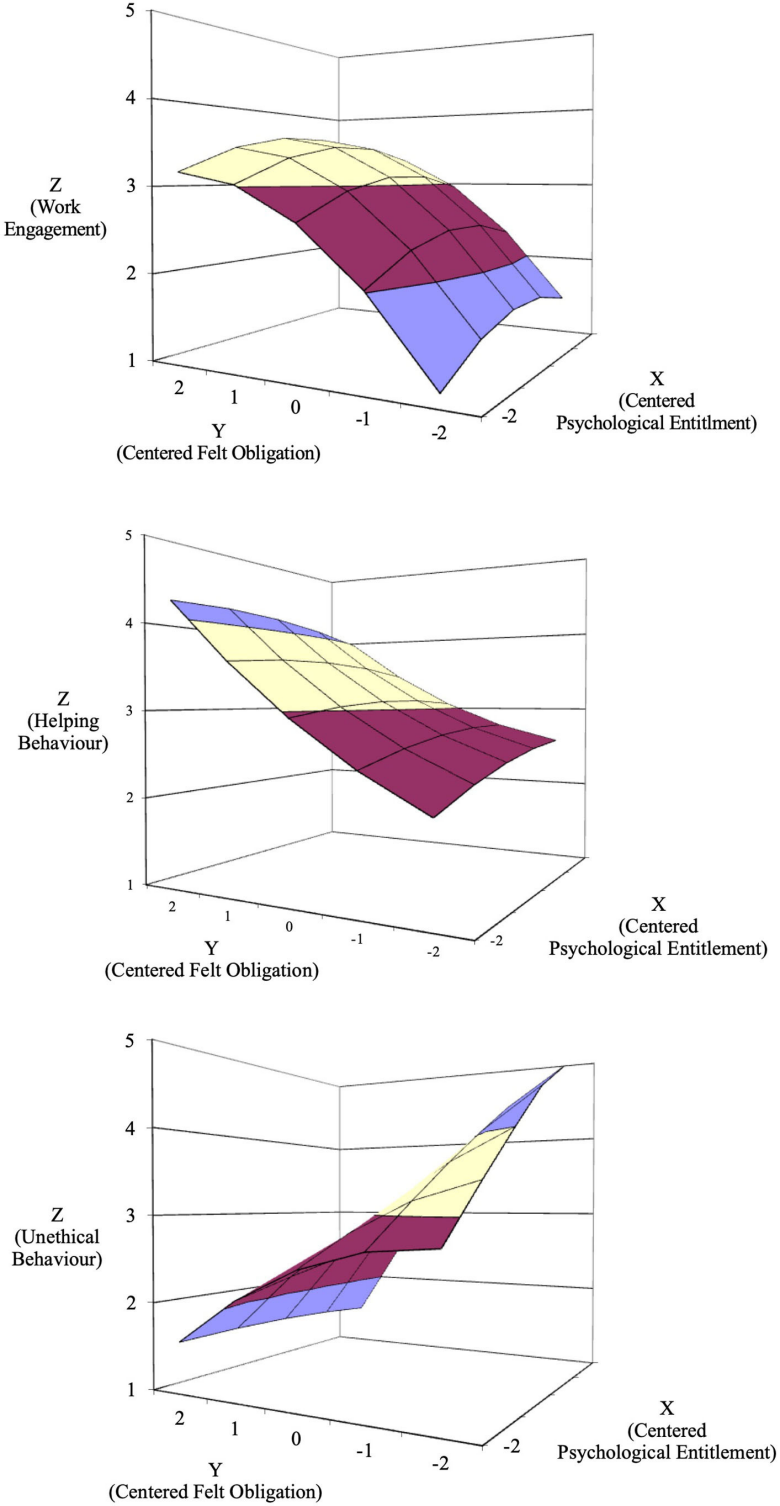
Response surface analysis results.

The results of Model 6 (Table 3) displayed that psychological entitlement was significantly positively associated with unethical behavior (*B* = 0.30, SE = 0.05, *p* < 0.01) while felt obligation was significantly negatively associated with unethical behavior (*B* = −0.60, SE = 0.06, *p* < 0.01). The results of Model 6 in Table 3 showed that the slope (β = −0.31, SE = 0.10, *p* < 0.01) for the congruence line (*x* = *y*) was negative and significant. The significantly negative slope indicated that, compared with independent employees, those interdependent employees had a lower level of helping behavior, thus supporting H2c. The results of Model 6 in Table 3 showed that the slope (β = 0.90, *SE* = 0.08, *p* < 0.01) for the incongruence line (*x* = –*y*) was significant. This result indicated that the level of unethical behavior was lower for other-oriented employees than for self-oriented employees, thereby confirming H2c. The results of the response surface analysis are shown in Figure 3.

**Figure 4:**
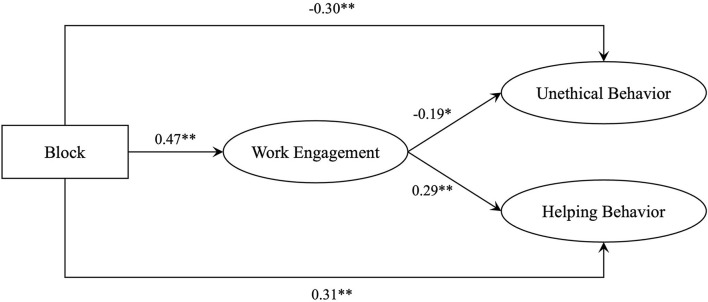
Results for the model. *p < 0.05 and **p < 0.01.

To examine the underlying mechanism linking the fit between psychological entitlement and felt obligation with (un)ethical behavior, this study used a block variable approach to test hypothesis 3 (Figure 4), with the results shown in Figure 4. The fit between psychological entitlement and felt obligation was positively correlated with work engagement (*B* = 0.47, *p* < 0.01). In addition, work engagement was positively associated with helping behavior (*B* = 0.29, *p* < 0.01) but negatively associated with unethical behavior (*B* = −0.19, *p* < 0.05).

To further test H3a and H3b, bootstrapping analysis was used to examine the direct and indirect effects (Table 4). For the role of work engagement in mediating the relationship between (in)congruences in psychological entitlement and felt obligation, and helping behavior, the indirect effect was significant [Effect = 0.22, SE = 0.09, 95% CI = (0.07, 0.41)], thereby confirming H3a. For the role of work engagement in mediating the relationship between (in)congruences in psychological entitlement and felt obligation, and unethical behavior, the indirect effect was significant [Effect = −0.24, SE = 0.10, 95% CI = (−0.47, −0.06)], thus verifying H3b.

**Table 4:** Results of bootstrapping analysis.

	**Effect**	**SE**	**95% confidence interval**	
			**UL**	**LL**
Block → Work engagement → Helping behavior	0.22	0.09	0.07	0.41
Block → Work engagement → Unethical behavior	−0.24	0.1	−0.47	−0.06

Bootstrapping = 20,000; LL, lower level; UL, upper level.

## 5. Discussion

This study has adopted a two-wave multi-source questionnaire survey to test the influence of the orthogonal structure of felt obligation and psychological entitlement on (un)ethical behavior. Based on the polynomial regression with response surface analysis, this study has found that interdependent employees have higher levels of work engagement, helping behavior, and unethical behavior compared with independent employees alongside the congruence line. Other-oriented employees have higher levels of work engagement, helping behavior, and unethical behavior compared with self-interested employees. Moreover, work engagement mediates the influence of fit between psychological entitlement and felt obligation on both helping behavior and unethical behavior. Our study has made two contributions to research on both psychological entitlement and felt obligation.

First, this study has explored the joint influence of psychological entitlement and felt obligation on employees' behavioral choices. Prior studies regarded psychological entitlement and felt obligation as two ends of one concept. For illustration, Brummel and Parker (2015) suggested that felt obligation and psychological entitlement are two distinct concepts. In fact, they both separately and jointly shape employees' behavioral choices. Existing studies predominantly focused on how psychological entitlement and felt obligation impacted individuals' behavior independently (Lorinkova and Perry, 2019; Alnaimi and Rjoub, 2021). To acquire some insights, this study has conducted empirical research to explore the joint influence of entitlement and obligation on employees' engagement and (un)ethical behavior based on the orthogonal structure of felt obligation and psychological entitlement proposed by Brummel and Parker (2015).

In terms of the incongruence line, this research has found that other-oriented employees with a higher obligation but a lower entitlement have higher levels of work engagement, helping behavior, and unethical behavior than self-oriented employees with a lower obligation but a higher entitlement. The results are consistent with prior studies highlighting the prosocial tendencies of felt obligation (Lee et al., 2019b) and self-serving tendencies of psychological entitlement (Neville and Fisk, 2019) within the theoretical framework of the social interdependence theory. In terms of the congruence line, this research has challenged the prior studies emphasizing the negative effect of high psychological entitlement on engagement and (un)ethical behavior. By virtue of the equity theory, this study has noted that obligated and entitled employees are motivated to devote more resources to distinguish themselves from their peers (Tennent, 2021). As a result, entitled employees tend to exhibit high engagement and helping behavior but low unethical behavior when they also have a high felt obligation to enhance the sense of equity. Arguably, this study has provided a novel insight into the outcomes of felt obligation and psychological entitlement.

Second, this study has uncovered the underlying path linking the fit between psychological entitlement and felt obligation with (un)ethical behavior. Work engagement was adopted by prior studies as an important mechanism to explain the influences of personality on ethical behavioral choices (Tisu et al., 2020). From the perspective of the equity theory, work engagement reflects the responses to their perception of their inputs to outputs at work, which further determines their ethical behavior (Agarwal, 2014). From a social interdependence perspective, work engagement reflects employees' commitment to contributing to organizational effectiveness by helping co-workers and inhibiting unethical behavior (Tjosvold et al., 2008). Although prior studies explored the antecedents and outcomes of work engagement, scarce research adopted work engagement to clarify the relationship between personality traits and (un)ethical behavior. Against this background, the present study has enjoyed novelty by adopting work engagement as a mediator to explain how (in)congruences in psychological entitlement and felt obligation impact employees' helping behavior and unethical behavior from both the equity theory and social interdependence theory perspectives.

This research has several practical implications for practitioners. Our findings suggest that entitled employees may be less likely to engage in their jobs and helping behavior, but more likely to engage in unethical behavior. Therefore, organizations should ensure they adopt strategies to reduce the likelihood of employees experiencing such unfavorable psychological states. For example, organizations may seek to measure psychological entitlement among their employees in selection and performance evaluation procedures and to identify employees with high entitlement.

However, for the companies which have recruited entitled employees, studies have not provided specific managerial strategies. This study finds that entitled employees also have outstanding performance when they have a high felt obligation, Therefore, several strategies can be adopted to stimulate entitled employees' work engagement and helping behavior and attenuate unethical behavior. According to existing studies, positive leadership and organizational support can be used to nurture felt obligation (Lorinkova and Perry, 2019; Thompson et al., 2020).

## 6. Limitations and future research

This study has several limitations, which help point out directions for future research. First, this study cannot establish causal relationship between focal variables. The adopted questionnaire survey design can only provide us with insights into the association between focal variables rather than the causal influences of psychological entitlement and felt obligation on behavioral choices. Future research may adopt a cross-lagged panel design to overcome these shortages.

Second, this study mainly focuses on the mediating effect of work engagement. There may be other alternative mechanisms that can explain the indirect influence of fit between psychological entitlement and felt obligation on (un)ethical behavior. For instance, organizational identification can also be used to explain the indirect relationship between personality and (un)ethical behavior. Future research may explore other mechanisms to enrich the present study.

Third, this study is conducted within Chinese culture. The entitlement and obligation vary with age and culture (Brummel and Parker, 2015). Future studies may replicate this study in a Western culture context to confirm its external validity.

## 7. Conclusion

By adopting two-wave multi-source data in leader-subordinate dyads, this study has explored the impacts of (in)congruences in entitlement and obligation on (un)ethical behavior. This research has found that when psychological entitlement and felt obligation are balanced at higher levels rather than lower levels, employees have higher work engagement and helping behavior but lower unethical behavior. By contrast, when psychological entitlement and felt obligation are asymmetric, employees with high felt obligation but low psychological entitlement have higher helping behavior and work engagement but lower unethical behavior compared with employees with low felt obligation but high psychological entitlement. In addition, work engagement plays a role in mediating the relationship between obligation–entitlement fit and (un)ethical behavior. Drawing on the social interdependence theory, this study has clarified the advantages of felt obligation and the disadvantages of psychological entitlement along the incongruence line, which is consistent with prior studies. Moreover, based on the equity theory, this study has proposed that high entitlement is beneficial to employees when their obligation is high simultaneously, enriching traditional entitlement literature. Through polynomial regression with response surface analysis, this study has provided a novel perspective to expand on the influences of psychological entitlement and felt obligation on ethical behavioral decision-making.

## Data availability statement

The original contributions presented in the study are included in the article/supplementary material, further inquiries can be directed to the corresponding authors.

## Author contributions

All authors listed have made a substantial, direct, and intellectual contribution to the work and approved it for publication.
